# Low Detection Rate of H3K27M Mutations in Cerebrospinal Fluid Obtained from Lumbar Puncture in Newly Diagnosed Diffuse Midline Gliomas

**DOI:** 10.3390/diagnostics11040681

**Published:** 2021-04-09

**Authors:** Jotaro On, Manabu Natsumeda, Jun Watanabe, Shoji Saito, Yu Kanemaru, Hideaki Abe, Yoshihiro Tsukamoto, Masayasu Okada, Makoto Oishi, Junichi Yoshimura, Akiyoshi Kakita, Yukihiko Fujii

**Affiliations:** 1 Department of Neurosurgery, Brain Research Institute, Niigata University, Niigata 951-8585, Japan; jotaro-on_silver@sky.hi-ho.ne.jp (J.O.); watanabejun1003@yahoo.co.jp (J.W.); sho-2.s@bri.niigata-u.ac.jp (S.S.); yu.k93@bri.niigata-u.ac.jp (Y.K.); hideabe04161983@gmail.com (H.A.); yoshi.tsukamoto@me.com (Y.T.); masayasu_okd@bri.niigata-u.ac.jp (M.O.); mac.oishi@mac.com (M.O.); junyoshi@bri.niigata-u.ac.jp (J.Y.); yfujii@bri.niigata-u.ac.jp (Y.F.); 2 Department of Pathology, Brain Research Institute, Niigata University, Niigata 951-8585, Japan; kakita@bri.niigata-u.ac.jp

**Keywords:** liquid biopsy, H3K27M-mutant, diffuse midline glioma, circulating tumor DNA, diagnosis

## Abstract

Recent studies have suggested the feasibility of detecting H3K27M mutations in the cerebrospinal fluid of diffuse midline glioma (DMG) patients. However, cerebrospinal fluid from patients in these studies were collected mainly during biopsy, ventriculo-peritoneal shunt procedures or postmortem. We assessed circulating tumor DNA (ctDNA) extracted from cerebrospinal fluid (CSF) and plasma in a series of 12 radiographically suspected and/or pathologically confirmed diffuse midline glioma patients and assessed for *H3F3A* K27M mutation using digital droplet PCR. In 10 patients, CSF was obtained by lumbar puncture at presentation. A definitive detection of *H3F3A* K27M mutation was achieved in only one case (10%); *H3F3A* K27M mutation was suspected in three other cases (30%). *H3F3A* K27M mutation was detected in two patients in CSF obtained by ventricular tap during a ventriculo-peritoneal shunt for obstructive hydrocephalus. Cases in which a definitive assessment was possible (definite *H3F3A* K27M or definite *H3F3A* wildtype) tended to be younger (median 7.5 years vs. 40.5 years; *p* = 0.07) and have a higher concentration of CSF protein (median 123 mg/dL vs. 27.5 mg/dL; *p* = 0.21) compared to nondefinite cases. Low proliferation and apoptotic rates seemed to be characteristics of DMG unfavorable for liquid biopsy. More advanced lesions with necrosis and evidence of dissemination were unlikely to be candidates for lumbar puncture due to the fear of exacerbating obstructive hydrocephalus. Methods to safely sample CSF and a more sensitive detection of ctDNA are necessary for reliable liquid biopsy of DMG at presentation.

## 1. Introduction

Recurrent *HIST1H3B* (H3.1K27M) or *H3F3A* (H3.3K27M) have been reported in 50 to 80% of diffuse intrinsic pontine gliomas and thalamic gliomas [1,2,3,4,5,6]; thus, the entity “diffuse midline gliomas, H3K27M-mutant” has been introduced in the revised fourth edition of World Health Organization Classification Tumours of the Central Nervous System (WHO2016) [7]. The discovery of these diagnostic, genetic abnormalities has led to excitement about noninvasive diagnosis by liquid biopsy of diffuse midline gliomas (DMGs), in which radical resection is often not possible due to localization to the brainstem and thalamus.

We have previously reported a 100% match of the *MYD88* mutation status in circulating tumor DNA (ctDNA) extracted from cerebrospinal fluid (CSF) and biopsied tissue in 21 primary and secondary central nervous system lymphoma patients using droplet digital PCR (ddPCR) [8]. Recent papers have suggested the feasibility of detecting H3K27M mutations in CSF by ddPCR [9,10] or next generation sequencing panel analysis [11] of ctDNA, giving the impression that a liquid biopsy for the diagnostic detection of H3K27M mutations in DMGs is feasible before the initiation of radiation treatment. However, a closer look at the papers reveals that CSF collected from patients in these studies mainly occurred during biopsy, ventriculo-peritoneal shunting or postmortem. In the present study, we shed light on the prospects of liquid biopsy of DMG at presentation and discuss what characteristics of brain tumors make the reliable detection of ctDNA in CSF possible.

## 2. Materials and Methods

Twelve patients with radiographically suspected diffuse midline glioma, treated at the Department of Neurosurgery, Niigata University Hospital between November 2017 and January 2021, who underwent liquid biopsy, were enrolled in the study. Written consent was obtained from patients and families for liquid biopsy after approval from the institutional review board (#G2018-0008). Detailed methods of ddPCR have been previously published [8]. Briefly, 1 mL of cerebrospinal fluid was obtained and centrifuged at 4 °C, 1000× *g* for 10 minutes to remove cells, and the supernatant was stored at −80 °C within 30 minutes of collection. ctDNA was extracted using the Maxwell RSC ccfDNA Plasma Kit (RSC; Promega, Leiden, The Netherlands), according to the manufacturer’s instructions. For all samples, DNA was dissolved in 60 μL of elution buffer and stored at −20 °C until further use. ddPCR reagents and primer/probe mix for *H3F3A* K27M were purchased from Bio-Rad (Hercules, CA, USA). A 20 μL PCR mix, composed of 10 μL 2× ddPCR Supermix for Probes (no deoxyuridine triphosphate; Bio-Rad), 1 μL ddPCR Mutation Assay (Bio-Rad) and 9 μL ctDNA, was loaded into sample wells of an eight-channel disposable droplet generator cartridge (Bio-Rad). An additional 70 μL of droplet generation oil (Bio-Rad) was loaded into the oil well for each channel. After droplet generation, the droplets were transferred into a 96-well PCR plate and subjected to thermal cycling. Amplification was carried out on the 20 μL reaction mixture on the QX-200 ddPCR system (Bio-Rad). After PCR, the 96-well PCR plate was subjected to the QX-200 droplet reader (Bio-Rad), and data were analyzed by QuantaSoft analysis software (Bio-Rad). *H3F3A* K27M-mutation specific signals were generated in the hexachloro-fluorescein channel. We considered definite mutant cases to have a fractional abundance of 0.1% or more and to have three or more mutant droplets and/or wildtype droplets detected. Cases in which only one or two mutant droplets were detected were considered H3K27M-mutant-suspect. Cases where two or fewer wildtype droplets were detected were considered unreliable. ddPCR was performed in at least two separate wells for each case whenever possible. Surgical removal or biopsy was performed in two cases (cases #7 and #11) with genetic confirmation of *H3F3A* K27M.

Comparisons between two groups were carried out using the Mann–Whitney *U* test and tests for associations between different parameters by the Fisher’s exact test for 2 × 2 contingency tables. Statistical analyses were performed using GraphPad Prism 9 software (GraphPad Software, http://www.graphpad.com accessed on 6 April 2021). *p* < 0.05 was considered significant.

## 3. Results

### 3.1. Patient Characteristics

Lesions were located at the pons in eight patients, medulla oblongata in one patient, thalamus in two patients and temporal lobe in one patient (Table 1). A liquid biopsy was performed a total of 19 times in 12 patients: at presentation in 10 patients, during adjuvant treatment in one patient, at recurrence in three patients and during ventriculo-peritoneal shunting in four patients. In one patient, a liquid biopsy was performed at presentation using both CSF and plasma. CSF was obtained by lumbar puncture in 10 patients, ventricular tap in three patients, and plasma was obtained from four patients. Pre- and post-contrast MR imaging was performed in all patients. Contrast enhancement was observed in 10 patients at presentation, and in one patient contrast enhancement was not observed at presentation but at relapse. Necrosis was observed in three patients at presentation and in one patient at relapse. Evidence of leptomeningeal disease was observed in three patients.

### 3.2. Detection Rate of H3F3A K27M at Presentation

**Figure 1:**
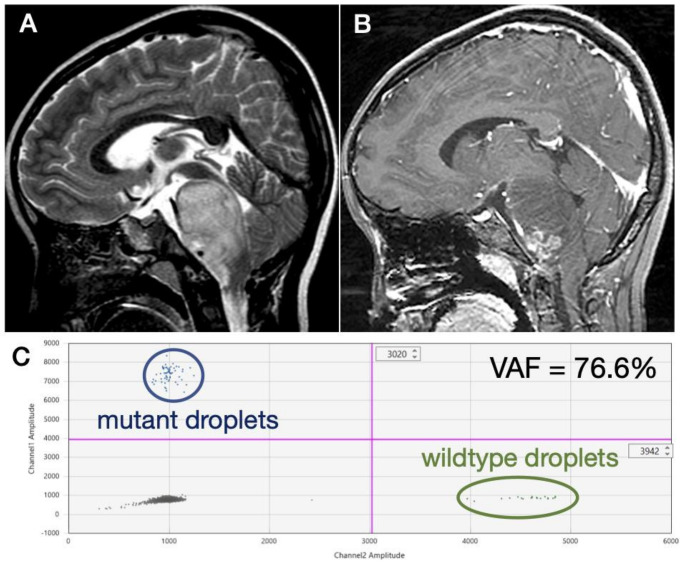
Representative case in which the *H3F3A* mutation was detected in CSF obtained by lumbar puncture at presentation. MR images show (**A**) a diffuse hyperintensity of the pons on T2-weighted images and (**B**) an exophytic growth at the lower pons and encasement of vertebral arteries by enhancing tumor on post-contrast images. (**C**) Many mutant and wildtype droplets were detected, and the variant allele frequency was 76.6%.

Lumbar puncture was performed in 10 patients at presentation (Table 1). However, a definite detection of the *H3F3A* K27M mutation was obtained in only one (10%) patient showing diffuse hyperintensity of the pons on T2-weighted MR images (Figure 1A) and an exophytic growth at the lower pons with encasement of vertebral arteries by enhancing tumor on post-contrast images (Figure 1B). A high variant allele frequency (VAF) of 76.6% was detected (Figure 1C). Exophytic growth at the lower pons and encasement of the vertebral arteries but not hydrocephalus was observed in this case. *H3F3A* K27M was suspected in three (30%) other patients at presentation, but only one or two mutant droplets were detected from each well in these patients (Table 2). Two cases were wildtype for *H3F3A*, in which wildtype droplets but not mutant droplets were observed. In three (30%) cases, the ddPCR results were considered unreliable, with a sufficient number of mutant and wildtype droplets not being detected.

### 3.3. Detection Rate of H3F3A K27M at Recurrence

**Figure 2:**
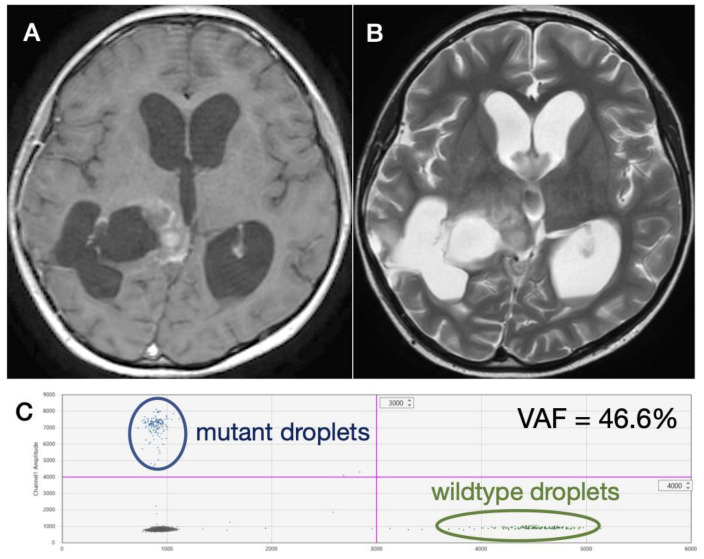
A recurrent DMG case in which *H3F3A* K27M was detected in CSF obtained during ventriculo-peritoneal shunting. MR images show (**A**) a heterogeneously enhancing residual lesion of the right thalamus and (**B**) enlarged ventricles. (**C**) Multiple *H3F3A* K27M-mutant droplets were detected by ddPCR analysis.

**Figure 3:**
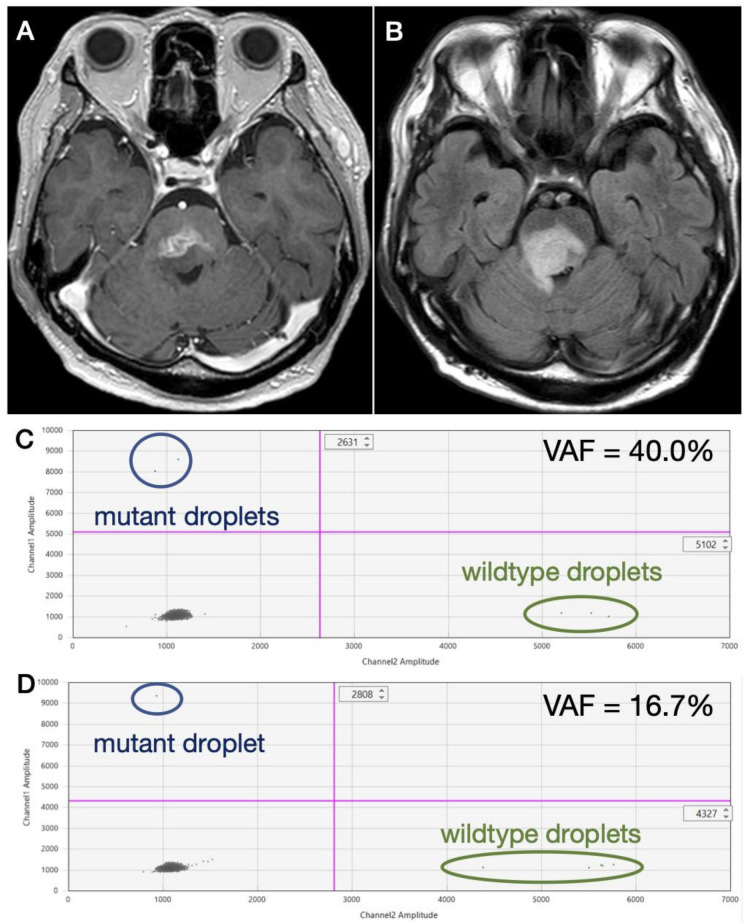
A recurrent DMG case in which *H3F3A* K27M was suspected. MR images show (**A**) a heterogeneously enhancing lesion with possible necrosis and (**B**) perifocal hyperintensity on fluid attenuated inversion recovery (FLAIR). Since mutant droplets were detected from both wells by ddPCR analysis, *H3F3A* K27M mutation was strongly considered. However, since only (**C**) two and (**D**) one mutant droplets were detected from each well, a definitive diagnosis was not achieved.

A definitive detection of *H3F3A* K27M was obtained at recurrence from two patients, both in CSF obtained from ventricular tap during ventriculo-peritoneal shunting for obstructive hydrocephalus in a recurrent DMG case with heterogeneously enhancing residual lesion of the right thalamus (Figure 2A) and enlarged ventricles (Figure 2B). Multiple *H3F3A* K27M-mutant droplets were detected by ddPCR analysis (Figure 2C). However, a definitive detection of *H3F3A* K27M was not achieved in patients undergoing lumbar puncture at recurrence in a DMG patient showing a heterogeneously enhancing lesion with possible necrosis (Figure 3A) and perifocal hyperintensity on fluid attenuated inversion recovery (FLAIR) (Figure 3B). Since mutant droplets were detected from both wells by ddPCR analysis, *H3F3A* K27M mutation was strongly considered (Figure 3C,D).

### 3.4. Comparison of Definite versus Nondefinite Cases

We compared the clinical, radiographical and CSF characteristics of cases in which there was a definite diagnosis (defined as either a definite *H3F3A* K27M mutation or definite *H3F3A* K27 wildtype) versus nondefinite (defined as *H3F3A* K27M-suspect or unreliable) cases (Table 3). Due to the lack of sample size, none of the analyzed factors yielded significant *p*-values, but there was a tendency for definite cases to be younger (median 7.5 years vs. 40.5 years; *p* = 0.07) and to have a higher concentration of CSF protein (median 123 mg/dL vs. 27.5 mg/dL; *p* = 0.21).

## 4. Discussion

Recurrent *HIST1H3B* (H3.1K27M) or *H3F3A* (H3.3K27M) mutations have been reported in 50 to 80% of diffuse intrinsic pontine gliomas and thalamic gliomas [1,2,3,4,5,6]. Thus, the noninvasive diagnosis of DMGs through the detection of circulating H3K27M mutations in CSF and plasma at presentation is highly anticipated.

Three previous reports highlight the feasibility of detecting H3K27M mutations in DMG patients by ctDNA analysis in CSF and plasma [9,10,11]. However, taking a closer look at the results reveals that the detection of H3K27M mutations in CSF obtained by lumbar puncture at presentation is still highly challenging. Huang et al. detected *HIST1H3B* K27M mutations in four out of six (66.7%) diffuse midline gliomas. In that study, CSF was either obtained from the ventricles by external ventricular drainage at tumor biopsy (*n* = 2), at reservoir placement (*n* = 2) or by reservoir tap during treatment (*n* = 2). Thus, CSF was not collected by lumbar tap before treatment in any of the cases. Subsequently, two large studies were reported. Pan et al. reported the detection of *H3F3A* K27M mutations (*n* = 27) or *HIST1H3B* K27M mutations (*n* = 5) in 32 out of 57 (56%) DMG patients by next generation sequencing (NGS). CSF was obtained from the cisterns during biopsy in 52 (91.2%) cases, from during ventriculo-peritoneal shunt procedures in two (3.5%) cases, and by lumbar puncture in only three (5.7%) cases [11]. Finally, Nazarian’s group found H3K27M mutations in 42 out of 48 (88%) subjects with DMGs. CSF was obtained from 28 patients, postmortem in 22 patients (78.5%) and upfront in only four patients (14.2%) [10].

An important study by Miller et al. detected ctDNA in CSF obtained by lumbar puncture in 42 out of 85 (49.5%) patients harboring WHO grade 2, 3 and 4 gliomas using NGS. ctDNA was detected from a higher percentage of grade 4 tumors (64% to 44%), in patients with a positive CSF cytology (18% vs. 0%), higher CSF protein concentration (72 vs. 56), higher number of prior resections (two vs. one), in recurrent cases (55% vs. 19%) and in larger-volume tumors (1553 mm^2^ vs. 373 mm^2^) [12]. Additionally, ctDNA has been detected from plasma in 55% of glioblastoma patients using the Guardant 360^R^ technology [13].

We have previously reported a high detection rate of *MYD88* L265P mutations in CSF in primary and secondary CNS lymphoma patients using ddPCR, with a 100% match of the *MYD88* L265P mutation status between tumor and CSF in 21 cases with available tumor samples [8]. A high VAF was detected in the majority of cases. By immediately centrifuging to remove cells and storing in −80 °C after obtaining CSF and other body fluids, we were even able to detect *MYD88* L265P mutations from the plasma and urine of PCNSL patients [14]. Since ctDNA is shed from cells undergoing apoptosis, tumors such as lymphomas with a rapid turnover may be easier to detect. The presence of lymphoglandular bodies, which are considered to reflect single cell apoptosis, is characteristic for PCNSL. Furthermore, a high lactate/lipid peak is often observed by magnetic resonance spectroscopy in PCNSL [15], reflecting rapid apoptosis. Furthermore, a marked contrast enhancement by Gadolinium, deep-seated location and frequent spinal dissemination are all features of PCNSL that favor the reliable detection of ctDNA. On the other hand, contrary to their dismal prognoses and midline location [16], DMGs are known to be slow growing [17,18], frequently show only a subtle contrast enhancement, and often lack leptomeningeal disease and necrosis at presentation [19].

The limitations of the present study include the small sample size, low number of pathologically confirmed cases and inability to detect *HIST1H3B* K27M mutations [3] due to the lack of a reliable ddPCR primer. We have previously employed the same methods in PCNSL and achieved a high detection rate of *MYD88* L265P and a 100% match of the *MYD88* mutation status between CSF and tumor. Therefore, we can safely say that, at present, detecting *H3F3A* K27M in CSF obtained by lumbar puncture in DMG patients is difficult. Using the Maxwell RSC ccfDNA Plasma Kit, up to 4 mL of ctDNA can be extracted in CSF instead of the 1 mL used in the present study, potentially improving the detection of small amounts of ctDNA. However, obtaining large amounts of CSF can be challenging and possibly harmful in these patients. The development of more sensitive methods, including next generation sequencing or more sensitive PCR methods, is necessary for the reliable detection of *H3F3A* K27M in DMG patients before the commencement of treatment.

## 5. Conclusions

In the present report, we found that the detection rate of *H3F3A* K27M by ddPCR in CSF obtained by lumbar puncture in DMG patients at presentation is low. Diagnosis by liquid biopsy at presentation may be possible in younger patients with a high protein concentration in CSF or evidence of leptomeningeal disease. Liquid biopsy is an exciting alternative to biopsy in DMGs, but both case selection and the development of more sensitive methods are vital.

## Figures and Tables

**Table 1:** Summary of patient and imaging characteristics.

Case#	Age	Sex	Location	Timing of Liquid Biopsy	Type of Fluid	CSF Source	Assessment	Gd-Enhancement	Necrosis	Dissemination
1	7	F	Pons	At presentation	CSF	LP ^1^	H3K27M	+	+	+
2	5	F	Pons	At presentation	CSF	LP ^1^	WT ^2^	+	+	−
3	59	M	Pons	At presentation	CSF	LP ^1^	WT ^2^	+	-	−
4	9	M	Pons	At presentation	CSF	LP ^1^	WT ^2^	±	−	−
				At presentation	Plasma	N/A ^3^	H3K27M? ^4^	±	−	−
5	31	F	Pons	At presentation	CSF	LP ^1^	H3K27M? ^4^	±	−	+
6	64	M	Pons	At presentation	CSF	LP ^1^	H3K27M? ^4^	−	−	−
				At recurrence	CSF	LP ^1^	H3K27M? ^4^	+	±	−
7	23	M	T ^5^	At presentation	CSF	LP ^1^	H3K27M? ^4^	−	−	−
				At recurrence	Plasma	N/A ^3^	H3K27M? ^4^	−	−	−
				During VP shunt	CSF	Vent tap ^6^	H3K27M? ^4^	−	−	−
8	69	M	Thal ^7^	At presentation	CSF	LP ^1^	Unreliable	+	−	−
9	7	F	Pons	At presentation	CSF	LP ^1^	Unreliable	+	+	−
				During VP shunt	CSF	Vent tap ^6^	Unreliable	+	+	−
10	50	F	MO ^8^	At presentation	CSF	LP ^1^	Unreliable	+	−	−
11	6	F	Thal ^7^	During VP shunt	CSF	Vent tap ^6^	H3K27M	+	−	+
				At recurrence	Plasma	N/A ^3^	H3K27M? ^4^	+	−	+
12	8	F	Pons	During treatment	Plasma	N/A ^3^	WT ^2^	+	−	−
				At recurrence	Plasma	N/A ^3^	H3K27M	+	−	−

^1^ Lumbar puncture, ^2^ Wildtype, ^3^ Not applicable, ^4^ H3K27M suspect, ^5^ Temporal lobe, ^6^ Ventricular tap, ^7^ Thalamus, ^8^ Medulla oblongata.

**Table 2:** Summary of liquid biopsies.

Case#	Type of Fluid	CSF Source	DNA Conc (ng/mL)	Protein Conc (mg/dL)	CSF Cytology Class	Mut/WT1 VAF1(%)	Mut/WT2 VAF2(%)	Assessment
1	CSF	LP ^1^	228	69	I	62/19 (76.6)	50/12 (80.7)	H3K27M
2	CSF	LP ^1^	186	14	I	0/15 (0.0)	0/4 (0.0)	WT ^2^
3	CSF	LP ^1^	216	123	I	0/135 (0.0)	0/185 (0.0)	WT ^2^
4	CSF	LP ^1^	432	217	I	0/22 (0.0)	N/A ^3^	WT ^2^
	Plasma	N/A ^3^	468	N/A ^3^	N/A ^3^	2/69 (2.8)	N/A ^3^	H3K27M? ^4^
5	CSF	LP ^1^	264	19	I	0/7 (0.0)	1/21 (4.5)	H3K27M? ^4^
6	CSF	LP ^1^	216	32	I	1/0 (100)	2/1 (66.7)	H3K27M? ^4^
	CSF	LP ^1^	318	69	I	2/3 (40.0)	1/5 (16.7)	H3K27M? ^4^
7	CSF	LP ^1^	504	N/A ^3^	N/A ^3^	1/39 (2.5)	N/A ^3^	H3K27M? ^4^
	Plasma	N/A ^5^	366	N/A ^3^	N/A ^3^	1/37 (2.6)	N/A ^3^	H3K27M? ^4^
	CSF	Vent tap ^5^	90	N/A ^3^	N/A ^3^	2/12 (14.3)	1/9 (10.0)	H3K27M? ^4^
8	CSF	LP ^1^	216	N/A ^3^	N/A ^3^	0/0 (0.0)	0/0 (0.0)	Unreliable
9	CSF	LP ^1^	330	23	I	0/1 (0.0)	0/0 (0.0)	Unreliable
	CSF	Vent tap ^5^	306	N/A ^3^	N/A ^3^	0/0 (0.0)	0/0 (0.0)	Unreliable
10	CSF	LP ^1^	120	29	I	0/2 (0.0)	0/0 (0.0)	Unreliable
11	CSF	Vent tap ^5^	330	538	III	151/173 (46.6)	120/165 (42.0)	H3K27M
	Plasma	N/A ^3^	294	N/A ^3^	N/A ^3^	0/14 (0.0)	1/36 (2.8)	H3K27M? ^4^
12	Plasma	N/A ^3^	414	N/A ^3^	N/A ^3^	0/50 (0.0)	N/A ^3^	WT ^2^
	CSF	Vent tap ^5^	222	N/A ^3^	N/A ^3^	22/30 (42.3)	30/40 (42.9)	H3K27M

^1^ Lumbar puncture, ^2^ Wildtype, ^3^ Not applicable, ^4^ H3K27M suspect, ^5^ Ventricular tap.

**Table 3:** Characteristics of definite vs. nondefinite cases.

Characteristic	Definite	Non-Definite	*p*-Value
	(*n* = 6)	(*n* = 6)	
Age (y); median (range)	7.5 (5–59)	40.5 (7–69)	0.07
Sex			
Male; number (%)	2 (33)	3 (50)	>0.99
Female; number (%)	4 (67)	3 (50)	
**Location**			
Pons; number (%)	5 (83)	3 (50)	0.55
Other; number (%)	1 (17)	3 (50)	
**CSF characteristics**			
Protein; median (range)	123 (14–538)	27.5 (19–69)	0.21
Positive cytology; number (%)	1 (17)	0 (0)	>0.99
**Radiographical features**			
Gd-enhancement; number (%)	6 (100)	4 (67)	0.45
Necrosis; number (%)	2 (33)	1 (17)	>0.99
Leptomeningeal disease: number (%)	2 (33)	1 (17)	>0.99

## Data Availability

The data presented in this study are available upon reasonable request.
